# Spectrum of *TERT* promoter mutations and mechanisms of activation in thyroid cancer

**DOI:** 10.1002/cam4.2467

**Published:** 2019-08-13

**Authors:** Federica Panebianco, Alyaksandr V. Nikitski, Marina N. Nikiforova, Yuri E. Nikiforov

**Affiliations:** ^1^ Department of Pathology and Laboratory Medicine University of Pittsburgh School of Medicine Pittsburgh Pennsylvania

**Keywords:** copy number change, promoter activity, promoter mutation, *TERT* activation, thyroid cancer

## Abstract

**Background:**

Reactivation of telomerase reverse transcriptase (*TERT*) is an important event in cancer. Two hotspot mutations in the *TERT* promoter region, c.‐124C > T (C228T) and c.‐146C > T (C250T), occur in various cancer types including thyroid cancer. They generate de novo binding sites for E‐twenty‐six (ETS) transcription factors causing increased *TERT* transcription. The aim of this study was to search for novel *TERT* promoter mutations and additional mechanisms of *TERT* activation in thyroid cancer.

**Methods:**

We studied 198 papillary thyroid carcinomas (PTCs), 34 follicular thyroid carcinomas (FTCs), 40 Hürthle cell carcinomas (HCCs), 14 poorly differentiated/anaplastic thyroid carcinomas (PDTC/ATC), and 15 medullary thyroid carcinomas (MTCs) for mutations in an −424 bp to +64 bp region of *TERT*. The luciferase reporter assay was used to functionally characterize the identified alterations. Copy number variations (CNVs) in the *TERT* region were analyzed using TaqMan copy number assay and validated with fluorescence in situ hybridization (FISH).

**Results:**

We detected the hotspot c.‐124C > T and c.‐146C > T mutations in 7% PTC, 18% FTC, 25% HCC, and 86% PDTC/ATC. One PTC carried a c.‐124C > A mutation. Furthermore, we identified two novel mutations resulting in the formation of de novo ETS‐binding motifs: c.‐332C > T in one MTC and c.‐104_‐83dup in one PTC. These genetic alterations, as well as other detected mutations, led to a significant increase in *TERT* promoter activity when assayed using luciferase reporter system. In addition, 5% of thyroid tumors were found to have ≥3 copies of *TERT*.

**Conclusions:**

This study confirms the increased prevalence of *TERT* promoter mutations and CNV in advanced thyroid cancers and describes novel functional alterations in the *TERT* gene promoter, including a point mutation and small duplication. These mutations, as well as *TERT* copy number alterations, may represent an additional mechanism of *TERT* activation in thyroid cancer.

## Graphical abstract

Novel alterations in *TERT* promoter, including single‐nucleotide variants and duplications, lead to increased *TERT* promoter activity, highlighting additional mechanisms of *TERT* activation in thyroid cancer.

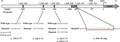

## 1 INTRODUCTION

Human telomerase reverse transcriptase (*TERT*) gene encodes the catalytic subunit of telomerase that, together with an RNA component, maintains chromosomal integrity by telomere elongation.^1^, ^2^ Telomerase is expressed in germline and stem cells, but it is normally repressed in postnatal somatic cells.^3^ Reactivation of *TERT* in cancer cells prevents telomere shortening, thus allowing unlimited cellular proliferation essential for transformation.^4^, ^5^ Telomerase activity is upregulated in ~85%‐90% of aggressive tumors, making this event a hallmark of cancer.^6^, ^7^

Somatic mutations in the promoter region of *TERT* are known to occur in cancer, providing a mechanism for *TERT* reactivation.^8^, ^9^, ^10^ Specifically, two recurrent mutations, located 124 bp and 146 bp upstream of the ATG translation start site and referred to as C228T (chr5, 1 295 228 C > T) and C250T (chr5, 1 295 250 C > T), have been reported in various cancers including thyroid cancer.^11^, ^12^, ^13^, ^14^ Both mutations stimulate *TERT* promoter transcription activity through the generation of de novo consensus binding sites “(T/A)TCC” for E‐twenty‐six (ETS) family of transcription factors, which in turn can be upregulated by MAPK signaling.^15^, ^16^, ^17^, ^18^, ^19^

Indeed, as reported in thyroid and other cancer types, *TERT* promoter mutations lead to an increase in *TERT* mRNA expression levels as compared to wild‐type (WT) *TERT*.^20^, ^21^, ^22^, ^23^, ^24^, ^25^

Other mechanisms of *TERT* reactivation include amplification of the *TERT* gene, as reported in some cancers, including follicular thyroid cancers.^23^, ^26^, ^27^, ^28^

Thyroid cancer is the most common type of endocrine tumors.^29^ The majority of thyroid tumors arise from thyroid follicular cells, comprising well‐differentiated thyroid papillary thyroid carcinoma (PTC) and follicular thyroid carcinoma (FTC), poorly differentiated thyroid carcinoma (PDTC) and anaplastic (undifferentiated) thyroid carcinoma (ATC).^30^ Initiation and progression of thyroid cancer occur through gradual accumulation of early and late genetic and epigenetic events, which lead to the activation of the MAPK and PI3K‐AKT signaling pathways.^31^, ^32^ Early genetic events in thyroid cancer progression (ie, *BRAF* mutations) are frequently found in both well‐differentiated thyroid cancer and in PDTC or ATC, therefore they are involved in tumor initiation and in the predisposition of the tumor to dedifferentiation. On the contrary, the late genetic events (ie, *TP53* mutations) occur with increasing frequency in tumors that progressively lose thyroid differentiation and therefore are associated with tumor progression and unfavorable outcome.^31^, ^33^ In thyroid cancer, *TERT* mutations represent a late event and are found in more aggressive thyroid cancers, being more common in PDTC and ATC (up to ~70% of cases) than in well‐differentiated PTC and FTC (~25%).^34^, ^35^ Moreover, *TERT* mutations have been established as an independent predictor of recurrence, distant metastases, poor prognosis, and cancer‐related mortality in well‐differentiated PTC and FTC.^36^, ^37^, ^38^, ^39^ Such mutations, C228T and C250T, have also been identified in Hürthle cell carcinoma (HCC) but not in medullary thyroid carcinoma (MTC).^34^ However, other regions of *TERT* promoter have not been fully characterized in thyroid tumors.

The aim of this study was to detect novel mechanisms of *TERT* activation in thyroid cancer by mutational screening of the extended promoter region of *TERT* and evaluating copy number variations (CNVs) involving the *TERT* chromosomal locus in different thyroid cancers.

## 2 MATERIALS AND METHODS

### 2.1 Tumor samples and cell lines

Snap‐frozen and formalin‐fixed paraffin‐embedded (FFPE) tissues from surgically removed thyroid samples and fine‐needle aspiration samples were collected at the Department of Pathology, University of Pittsburgh Medical Center and also obtained from the University of Pittsburgh Health Sciences Tissue Bank (HSTB). Study was done in accordance with the US Federal Policy for the Protection of Human Subjects and approved by the University of Pittsburgh Institutional Review Board. Thyroid cancer tissues were fixed with 4% phosphate‐buffered paraformaldehyde for 24 hours. Histologic sections were reviewed to confirm the diagnosis. Overall, 301 thyroid tumors were collected with following diagnosis: PTC (n = 198), FTC (n = 34), HCC (n = 40), PDTC/ATC (n = 14), and MTC (n = 15).

The following cell lines were used: normal thyroid HTori‐3 cells were purchased from European Tissue Culture Collection (ECACC) (cat# 90011609); follicular thyroid cancer FTC‐133 cells (Sigma/ECACC, cat# 94060901), papillary thyroid cancer K1cells (Sigma/ECACC, cat# 92030501), TPC‐1cells (Sigma cat# SCC147), and anaplastic thyroid cancer TTA1 cells were obtained from Dr Rebecca Schweppe (University of Colorado Cancer Center); anaplastic thyroid cancer SW1739 (Cell Lines Service, cat# 300453), C643 (Cell Lines Service, cat# 300298), and T241 cells were obtained from Dr Jeffrey Knauf and Dr James Fagin (Memorial Sloan Kettering Cancer Center).

### 2.2 DNA extraction

Genomic DNA was isolated from snap‐frozen and FFPE tissues using QIAmp DNA Mini Kit (Cat#51304, Qiagen) and QIAmp DNA FFPE Tissues Kit (Cat #56404, Qiagen), respectively.

### 2.3 Mutational analysis for *TERT* promoter

*TERT* promoter mutation status was determined using targeted polymerase chain reaction (PCR) amplification followed by Sanger sequencing of the −424 to +64 (relative to ATG) *TERT* promoter region (chr5: 1 295 528 to chr5: 1 295 040 (hg19). Briefly, PCR reactions were performed in a total reaction volume of 50 μL, containing 1X AmpliTaq Gold 360 Master Mix (Cat #4398881, Life Technologies), 10 μL of 360 GC enhancer, 0.2 μM of each primer, and ~60 ng of DNA template, using primers reported in Table S1. The PCR products were purified using MinElute PCR Purification kit (Cat #28006, Qiagen), and then sequenced by Sanger sequencing. Sanger sequencing was performed in both directions using the BigDye Terminator Kit v3.1 (Cat #4337456, Thermo) and an ABI 3130xl DNA Sequencer (Applied Biosystems).

### 2.4 Plasmids

*TERT* promoter regions encompassing −424 to −1 bp (relative to ATG) with WT sequence and with c.‐104_‐83dup mutation were synthesized and cloned into the multiple cloning site of pEZX‐GA01 Gaussia Luciferase (GLuc) and secreted alkaline phosphatase (SEAP) dual reporter vector (Cat #ZX103, GeneCopoeia) by GeneScript according to the company's protocols. Briefly, synthesized WT and c.‐104_‐83dup *TERT* promoters were inserted into pEZX‐GA01 vector upstream of GLuc reporter gene between EcoRI and HindIII sites. SEAP reporter was driven by a cytomegalovirus (CMV) promoter that was used as an internal control. To generate mutations corresponding to c.‐124C > T, c.‐146C > T, c.‐124C > A, c.‐332C > T, and c.‐104_‐83dup_Mut (c.‐104_‐83dup with eliminated ETS‐binding motifs: “TTCCTTTCC” changed to “TGCCTTTCA”) and the variant allele for rs2853669 polymorphism, site‐directed mutagenesis was then performed by GeneScript according to company's protocols using primers listed in Table S1.

### 2.5 Cell culture and luciferase reporter assay

For the luciferase reporter assay, HTori‐3, FTC‐133, and TTA‐1 cells were seeded at a density of 3‐4 × 10^5^ per well in 6‐well plates and transiently transfected the following day with 1 µg of each dual reporter construct using Lipofectamine 2000 (Cat #11668019, Invitrogen). Independent transfection experiments were performed six (for WT and c.‐104_‐83dup constructs) or three times (for rs2853669, c.‐124C > T, c.‐146C > T, c.‐124C > A, c.‐332C > T, and c.‐104_‐83dup_Mut constructs) on each cell line. After 24 hours, the culture medium was collected and stored at −80°C. The GLuc and SEAP activities were measured in triplicates per each experiment with the secreted‐pair dual luminescence kit (Cat #LF032, GeneCopoeia) using Spark™ 10M multimode microplate reader (Tecan) according to the manufacturer's instructions. The GLuc/SEAP luminescence ratios from each triplicate measurement were averaged and considered as transcriptional activity of *TERT* promoter variant per experiment. The transcriptional activity of each *TERT* promoter variant was normalized to the average (from six transfection experiments) transcriptional activity of WT *TERT* promoter in each cell line and defined as “normalized‐to‐WT transcriptional activity.”

### 2.6 TaqMan copy number assay

Copy number variations affecting the *TERT* gene were analyzed using the TaqMan copy number assay targeting intron 6 (ID: Hs06005815_cn; Applied Biosystems) on a 7500 Real‐Time PCR System (Applied Biosystems), according to the manufacturer's recommendations. RNase P TaqMan Copy Number Reference Assay (ID: 4403326; Applied Biosystems) was used for assay normalization. Briefly, all reactions were performed in quadruplicate in 20‐μL final volume containing 5‐ng DNA substrate, 10 μL of TaqMan Genotyping Master Mix (ID: 4371353, Applied Biosystems), 1 μL of TaqMan Copy Number Assay, which contains two primers and a carboxyfluorescein (FAM) dye‐labeled MGB probe to detect the genomic DNA target sequence, and 1 μL of TaqMan Copy Number Reference Assay, which contains two primers and a 2′‐chloro‐phenyl‐1,4‐dichloro‐6‐carboxyfluorescein (VIC) and TAMRA dye‐labeled probe to detect the genomic DNA reference sequence. The cycling conditions were: 95°C for 10 minutes, followed by 40 cycles each of 95°C for 15 seconds and 60°C for 1 minute. Average CT values were 24.73 to 30.117 for FAM and 25.46 to 33.63 for VIC. Raw data were analyzed for copy number using CopyCaller software (Applied Biosystems), as recommended. Briefly, CopyCaller Software performed relative quantification (RQ), following the comparative delta‐delta threshold cycle (ΔΔCT) method. Normal thyroid DNA samples were used as diploid controls.

### 2.7 Fluorescence in situ hybridization (FISH)

Copy number variations affecting the *TERT* gene were further validated using FISH. Tumor touch imprints were prepared from snap‐frozen tissues. For FISH probes generation, RP11‐990A6 (*TERT* gene 5p15.33 locus) and RP11‐461014 (reference 5q31 locus) BAC clones (BAC/PAC Resources, Children's Hospital Oakland Research Institute) were labeled by Nick translation kit (Cat #07j00‐001, Abbott Molecular) with SpectrumOrange‐dUTP (Cat # 02N33‐050, Abbott Molecular) and SpectrumGreen‐dUTP (Cat # 02N32‐050, Abbott Molecular), respectively. Hybridization was done as previously described.^40^ Microscopy was performed using Leica SP5 TCS confocal laser scanning microscope (Leica Microsystems) and images were captured using 63×, 1.4 NA oil PlanApo objective and 5× digital zoom.

### 2.8 Statistical analysis

Statistical analysis of the transcriptional activity of the WT and mutated *TERT* promoter regions was performed in pairs using Mann‐Whitney test in GraphPad Prism 6 (GraphPad Software Inc.). *P* were two‐sided and considered significant if <.05.

## 3 RESULTS

### 3.1 Detection of canonical and novel *TERT* promoter mutations in thyroid cancer

**Figure 1:**
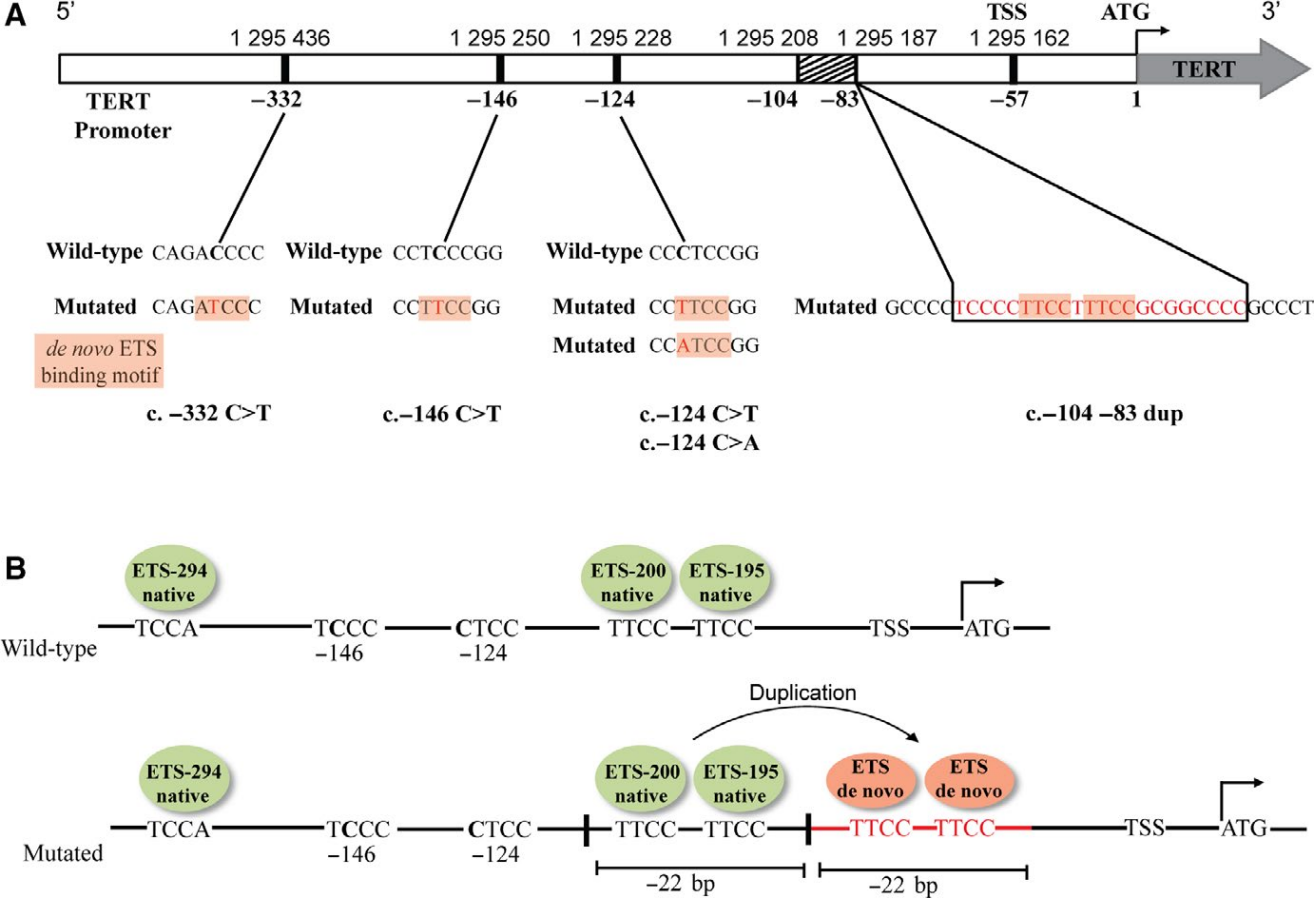
Schematic representation of mutations in *TERT* promoter region identified in thyroid tumors. A, Scheme of *TERT* promoter region with nucleotide numbering position of the known point mutations c.‐146C > T, c.‐124C > T, and c.‐124C > A, a novel point mutation c.‐332C > T, and a novel duplication at −104 to −83 relative to the ATG translation start site on chromosome 5 (chromosome positions are based on GRCh37/hg19) (TSS, transcription start site). The nucleotide substitution and the duplication in the mutant sequences are shown by red color. The wild‐type (WT) nucleotide corresponding to the point mutations is indicated in bold in the WT sequence. Each of these mutations leads to the generation of de novo binding motif for E‐twenty‐six (ETS) transcription factors (highlighted by pink‐color rectangles). B, Scheme of the duplication mutation. On the top, the WT* TERT* promoter sequence showing the native ETS binding motifs (in green) close to −146 and −124 hotspot positions that are indicated in bold; on the bottom, the mutant sequence showing the native and de novo putative ETS‐binding motifs (in pink) generated by c.‐104_‐83 duplication in *TERT* promoter

To search for novel *TERT* mutations, 301 thyroid tumor samples representing common types of thyroid cancers were subjected to Sanger sequencing analysis of the extended *TERT* promoter region (424 bp upstream and 64 bp downstream of the translation start site). As expected, the majority of the detected genetic alterations in the *TERT* promoter was presented by known c.‐124C > T (C228T) and c.‐146C > T (C250T) mutations. The most common c.‐124C > T *TERT* mutation was found in 10 (5%) PTC, 6 (17.6%) FTC, 10 (25%) HCC and 8 (57.1%) PDTC/ATC, but not in any MTC analyzed (Table 1; Figure S1A). In one (0.5%) PTC, the analysis showed a *TERT* mutation at the same hotspot position of −124 bp but with a different nucleotide substitution, c.‐124C > A. The second common mutation, c.‐146C > T, was detected in four (2%) PTC and four (28.6%) PDTC/ATC but not in any other tumors analyzed (Table 1; Figure S1A). Besides known *TERT* promoter mutations, we identified two novel mutations: c.‐332C > T and c.‐104_‐83dup (Table 1; Figure 1A; Figure S1B). The former is a substitution of cytosine‐to‐thymine at a −332 bp position (Chr 5:1 295 436 hg19 position) from the ATG initiation site. The tumor harboring this mutation was diagnosed as MTC and had no known hotspot *TERT* mutations. Similar to the hotspot mutations, the c.‐332C > T mutation generates the “ATCC” sequence, which is a de novo putative ETS‐transcription factor binding motif (Figure 1A).^15^, ^20^ The other mutation was found in one PTC and was a duplication of the 22‐bp *TERT* promoter region located −104 bp to −83 bp (Chr 5:1 295 208 to Chr 5:1 295 187 hg19 position) from ATG site. This tumor was also WT for hotspot *TERT* mutations. The duplication contained the sequences of the ETS‐195 and ETS‐200 motifs, resulting in the generation of two additional de novo “TTCC” ETS‐binding motifs (Figure 1B).^19^, ^20^ Therefore, in comparison to WT sequence, which harbors three native ETS motifs (ETS‐195 bp, ETS‐200 bp, and ETS‐294 bp), the mutant sequence was found to harbor five adjacent ETS‐binding sites, which include three native and two de novo sites (Figure 1B).

**Table 1:** Frequency of *TERT* promoter mutations in thyroid tumors

Thyroid tumor types	Number of cases studies	c.‐124C > T (C228T) n/N (%)	c.‐146C > T (C250T) n/N (%)	Other *TERT* mutations n/N (%)
PTC	198	10/198 (5%)	4/198 (2%)	c.‐104_‐83dup 1/198 (0.5%)
				c.‐124C > A 1/198 (0.5%)
FTC	34	6/34 (17.6%)	0/34 (0%)	—
HCC	40	10/40 (25%)	0/40 (0%)	—
PDTC/ATC	14	8/14 (57.1%)	4/14 (28.6%)	—
MTC	15	0/15 (0%)	0/15 (0%)	c.‐332C > T 1/15 (6.7%)

Abbreviations: FTC, follicular thyroid carcinoma; HCC, Hürthle cell carcinoma; MTC, medullary thyroid carcinoma; PDTC/ATC, poorly differentiated/anaplastic thyroid carcinoma; PTC, papillary thyroid carcinoma.

In addition to somatic mutations, we found several single‐nucleotide polymorphisms (SNP) in analyzed *TERT* promoter region in different thyroid tumor types. Specifically, we detected a common polymorphism rs2853669 in 50% of all thyroid tumors analyzed. We also identified four rare polymorphisms, rs35161420 and rs35226131, together in 5.6% of cases, and rs34233268 and rs347646448 each in 0.3% of cases studied (Table S2).

### 3.2 Functional characterization of novel *TERT* promoter mutations

**Figure 2:**
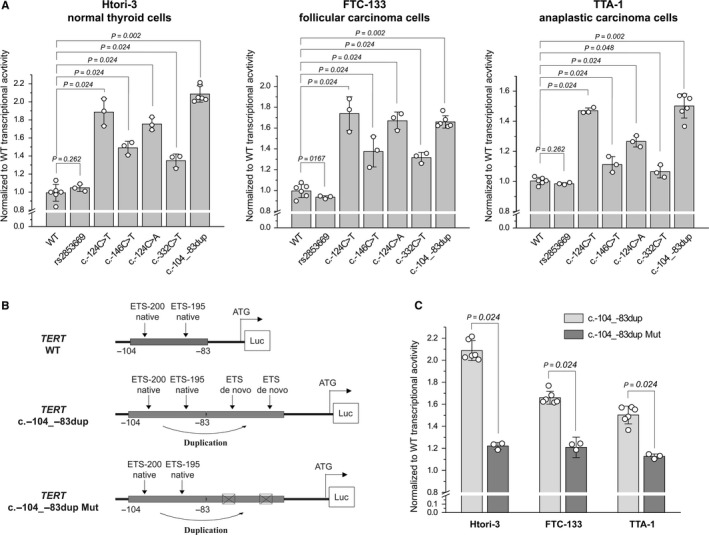
Effect of mutations on *TERT* promoter activity in thyroid cells. A, Graphs showing the transcriptional activity of *TERT* promoter region with wild‐type (WT) sequence, rs2853669T > C polymorphism, c.‐124C > T, c.‐146C > A, c.‐124C > A, c.‐332C > A, and c.‐104_‐83dup mutations in normal and cancer thyroid cell lines. Each dot represent normalized‐to‐WT transcriptional activity (average of three measurements) of the specific promoter variant assayed in six (for WT and c.‐104_‐83dup) or three (for rs2853669T > C, c.‐124C > T, c.‐146C > A, c.‐124C > A, and c.‐332C > A) independent experiments. Solid bars and whiskers represent mean and standard deviation, respectively. Statistical comparisons were performed using Mann‐Whitney test. B, Scheme of *TERT* promoter regions in luciferase reporter constructs showing location of native and de novo ETS‐binding motifs in WT and c.‐104_‐83dup luciferase reporter constructs, and elimination of de novo ETS sites in c.‐104_‐83dup Mut construct. C, Graph showing decreased transcriptional activity of *TERT* promoter region with c.‐104_‐83dup when ETS‐binding motif is inactivated by mutagenesis (c.‐104_‐83dup Mut). Each dot represent normalized‐to‐WT transcriptional activity (average of three measurements) of the specific promoter variant assayed in six (for c.‐104_‐83dup) or three (for c.‐104_‐83dup Mut) independent experiments. Solid bars and whiskers represent mean and standard deviation, respectively. Statistical comparisons were performed using Mann‐Whitney test

Next, we determined whether the newly identified *TERT* mutations are functional and lead to the increase in *TERT* promoter activity. To test this, the WT and all *TERT* promoter mutations found in this study (c.‐124C > T, c.‐146C > T, c.‐124C > A, c.‐332C > T, c.‐104_‐83dup) were assayed using a luciferase reporter assay in several thyroid cell lines. First, we genotyped several human thyroid cell lines for *TERT* promoter mutations and the majority of them were positive for mutations (Table S3). For luciferase assay, we used TTA1 (ATC) and HTori‐3 (normal human thyroid) cell lines with WT* TERT* promoter sequence, and FTC‐133 (follicular thyroid cancer) cell line carrying the c.‐124C > T mutation. As compared to the WT* TERT* promoter, all mutations conferred a significant up to twofold increase in transcriptional activity in all three thyroid cell lines examined, whereas the most common rs2853669 polymorphism did not alter the promoter activity as compared to the WT* TERT* sequence (Figure 2A). Among the mutated *TERT* promoters, the promoters with c.‐124C > T, c.‐124C > A, and c.‐104_‐83dup had the strongest transcriptional activities (Figure 2A).

Next, we explored whether the increased transcriptional activity of *TERT* promoter harboring the c‐104_‐83dup is related to the generation of two de novo ETS‐binding motifs. To test this, we eliminated the core of both motifs by site‐directed mutagenesis (Figure 2B). This significantly reduced the promoter transcriptional activity compared to the intact c.‐104_‐83dup mutation(Figure 2C). Taken together, these results suggest that all novel somatic mutations in the *TERT* promoter region are functional and lead to the increase in its transcriptional activity.

### 3.3 Copy number variations of *TERT* in thyroid cancer

**Figure 3:**
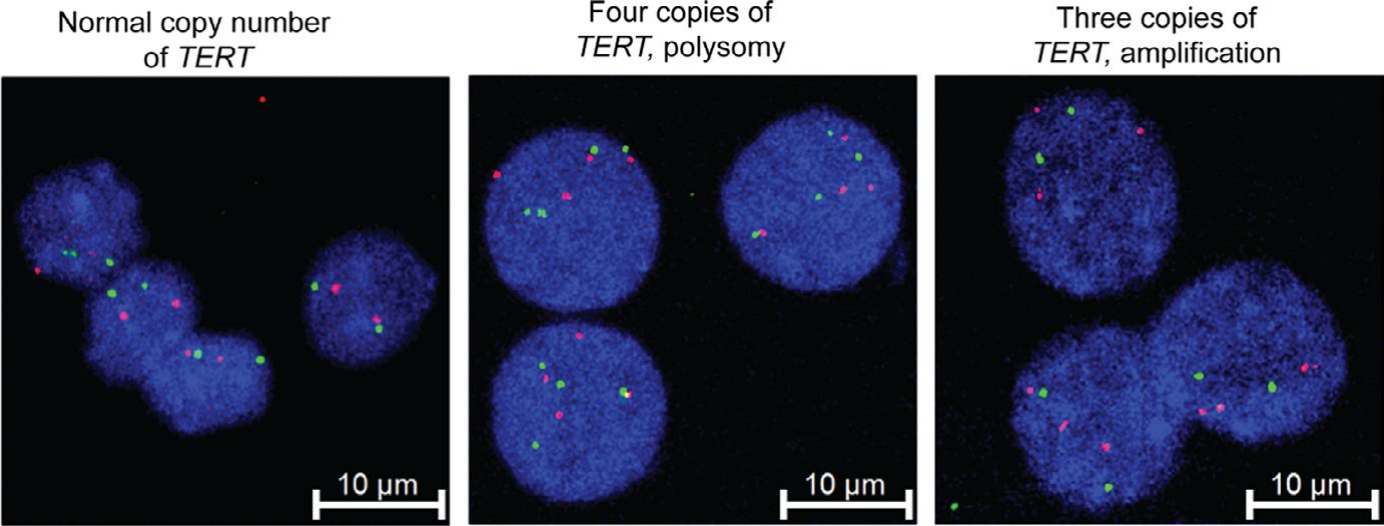
Validation of *TERT* numerical changes by fluorescent in situ hybridization (FISH). Representative FISH images showing normal or increased copy number of *TERT* *Left*: papillary thyroid carcinoma nuclei with diploid genotype showing cell nuclei containing two copies of *TERT* and 5q31 regions. *Central*: poorly differentiated carcinoma nuclei with chromosome 5 polysomy showing four copies of *TERT* and 5q.31 regions. *Right*: Hürthle cell carcinoma nuclei with *TERT* locus amplification showing three copies of *TERT* and two copies of 5q.31 regions. FISH signals: red represents *TERT* 5p15.33 locus and green represents 5q31 reference locus. Scale bars, 10 µm. Images taken using ×63 lens and ×5 digital zoom

In order to test whether CNV may represent another mechanism of *TERT* activation in thyroid cancer, DNA samples from 184 thyroid tumors were screened for CNV using a TaqMan copy number assay based on quantitative real‐time PCR (qPCR) (Table 2). Our analysis identified nine thyroid tumor samples with increased *TERT* locus copy number. Specifically, 1/107 PTC showed four copies of the *TERT* region, and 2/22 FTC, 4/29 HCC, and 2/4 PDTC/ATC showed three copies. None of the 22 MTCs analyzed showed numerical changes in the *TERT* locus (Table 2). Among the thyroid tumors with increased *TERT* gene copy numbers, 2/4 HCC samples and 1/2 PDTC/ATC samples were positive for c.‐124C > T mutation (Table 2). Several thyroid cell lines, including FTC133, K1, HTori‐3, T241, and TPC1, were also tested for *TERT* CNV and have not shown any alterations in the *TERT* copy number. To validate the qPCR CNV results, FISH was performed on available frozen samples from eight tumors with increased *TERT* locus copy number (a PTC with normal copy number of *TERT* was used as a diploid control*)*. FISH analysis confirmed the qPCR results and showed that increased copies of *TERT* were represented by the numerical change of the entire chromosome 5 or by amplification of the *TERT* locus (Figure 3). Only limited number of these samples had mRNA available, precluded the informative analysis of correlation between the copy number alterations, and expression levels of the *TERT* gene in these tumors.

**Table 2:** Copy number variation analysis using qRT‐PCR

Thyroid tumor types	Number of cases studied	Cases with increased *TERT* copy number (TaqMan copy number assay)	Cases harboring hotspot *TERT* mutations (among tumors with increased *TERT* copy number)
PTC	107	1/107 (0.9%)	0/1
FTC	22	2/22 (9.1%)	0/2
HCC	29	4/29 (13.8%)	2/4
MTC	22	0/22 (0%)	0
PDTC/ATC	4	2/4 (50%)	1/2

Abbreviations: FTC, follicular thyroid carcinoma; HCC, Hürthle cell carcinoma; MTC, medullary thyroid carcinoma; PDTC/ATC, poorly differentiated/anaplastic thyroid carcinoma; PTC, papillary thyroid carcinoma.

Analysis of *TERT* CNV in thyroid tumors by TaqMan copy number assay.

## 4 DISCUSSION

Reactivation of telomerase activity in cancer cells has been recognized as an important feature of cancer cells, although its mechanisms are not fully understood.^8^ A significant advance in this area was achieved after the discovery of recurrent *TERT* promoter mutations first in melanoma^11^, ^16^ and subsequently in many other human cancers including thyroid cancer.^14^ In thyroid cancer, multiple studies have found c.‐124C > T (C228T) and c.‐146C > T (C250T) *TERT* mutations in follicular cell‐derived thyroid cancers and particularly in PDTC and ATC.^14^, ^35^, ^38^ No *TERT* promoter mutations were previously documented in MTCs.^34^

The prevalence of the hotspot mutations, c.‐124C > T and c.‐146C > T, in our study is consistent with previous reports as it shows the lowest prevalence in PTC, followed by FTC and HCC, with the highest prevalence in PDC/ATC, whereas none of these mutations were seen in MTC. An additional mutation, c.‐124C > A, was also detected in one PTC. This mutation was already reported with low frequency in different cancer types 13, 20 including thyroid cancer, where it was also previously reported to occur in 0.3% of PTC.^41^

However, in this study, we report two novel mutations found using the analysis of an extended region of *TERT* promoter. The first mutation, c.‐332C > T, was detected in a single tumor case and was only one *TERT* promoter mutation in MTC group of tumors (1/15). The second novel mutation, c.‐104_‐83dup, was identified in one PTC case and was presented as duplication of 22 base pairs within the *TERT* promoter. Both c.‐332C > T and c.‐104_‐83dup mutations showed a stimulatory effect on transcriptional activity of *TERT* promoter in normal and cancer thyroid cells as we showed using luciferase reporter assay approach. The increased activity, if *TERT* promoter affected by these mutations, as well as other mutations detected in this study, is likely associated with the generation of de novo ETS‐transcription factor binding sites. Genetic alterations that create additional ETS‐binding sites in the vicinity of native sites are a known mechanism of aberrant *TERT* activation in cancer.^19^ In fact, Bell et al showed that c.‐124C > T and c.‐146C > T cooperate with native ETS sites, especially with ETS‐195 and ETS‐200, to recruit GA‐binding protein (GABP) transcription factor and to directly initiate the upregulation of *TERT* expression in glioblastoma.^19^ They also showed that, in addition to point mutation creating de novo ETS sites, a duplication event of 41‐bp generates one de novo ETS motif in one tumor. Similarly, the novel c.‐104_‐83dup creates two additional ETS motifs adjacent to three native sites. In addition, we showed that this is in a significant degree due to the two de novo ETS sites generated by the duplication. Indeed, elimination of the ETS sites in the duplicated region significantly reduced the promoter activity, although it did not bring it back to the WT level. Such residual activity is likely associated with the presence of binding motifs for other transcriptional factors and changes in the DNA helical turns.^19^, ^42^, ^43^ Due to the lack of RNA material from the tumor cases positive for new mutations, we have not tested whether the stimulatory effect of this mutation results in increased TERT mRNA level.

Reactivation of telomerase in human malignancies has also been linked to *TERT* amplification.^44^, ^45^ In fact, the *TERT* gene locus was found to be amplified in various cancers including lung, breast, cervical carcinomas, and FTC,^28^, ^45^, ^46^, ^47^ prompting us to examine the *TERT* gene for copy number alterations in different types of thyroid cancer. Interestingly, we observed that the increase in *TERT* locus copy number was more common in FTC, HCC, and PDTC, as compared to PTC. Moreover, *TERT* copy number gain co‐occurred with *TERT* hotspot mutations in some HCC and PDTC, suggesting that it may cooperate with hotspot mutations in *TERT* reactivation and cancer progression. Due to the unavailability of mRNA samples, we could not study directly the effects of CNV on *TERT* mRNA expression. However, other studies have shown the association between *TERT* copy number gain and mRNA expression in several cancer types, including FTC.^26^, ^28^, ^48^ Nevertheless, larger studies, particularly those focusing on advanced thyroid cancers, are required to provide definitive confirmation of this mechanism.

In summary, we report herein two novel *TERT* promoter alterations in thyroid tumors and show that these mutations are functional and lead to increase in the transcriptional activity of *TERT* promoter in thyroid cancer cells by creating new consensus motifs for transcriptional regulators. This suggests that when the promoter region of *TERT* is analyzed for mutations, it may need to include a more extended region, and otherwise some functionally relevant mutations may be missed. Our study also shows a higher prevalence of *TERT* copy number gains in aggressive thyroid cancer types, particularly in HCC and PDTC. Thus, novel functional mutations uncovered in this study and increased copy number of *TERT* may represent additional mechanisms of *TERT* activation in thyroid cancer.

## CONFLICT OF INTEREST

All authors have nothing to disclose.

## AUTHOR CONTRIBUTIONS

FP: Conceptualization, investigation, methodology, data analysis. AN: Investigation, methodology, data analysis. MN: Conceptualization, investigation. YN: Conceptualization, data analysis, project administration. All authors: Drafting of manuscript.

## SIGNIFICANCE

Novel functional mutations in *TERT* promoter, including single‐nucleotide variants and duplications, lead to increased *TERT* promoter activity, highlighting additional mechanisms of *TERT* activation in thyroid cancer.

## Data Availability

The data that support the findings of this study are available from the corresponding author upon reasonable request.
