# Supplementary material for: Spectrum of TERT promoter mutations and mechanisms of activation in thyroid cancer
Source: Cancer Med. 2019 Aug 13;8(13):5831–9. doi: 10.1002/cam4.2467 (PMC6792496; doi:10.1002/cam4.2467)
Supplement: Supplementary file 1 [file CAM4-8-5831-s001.docx]

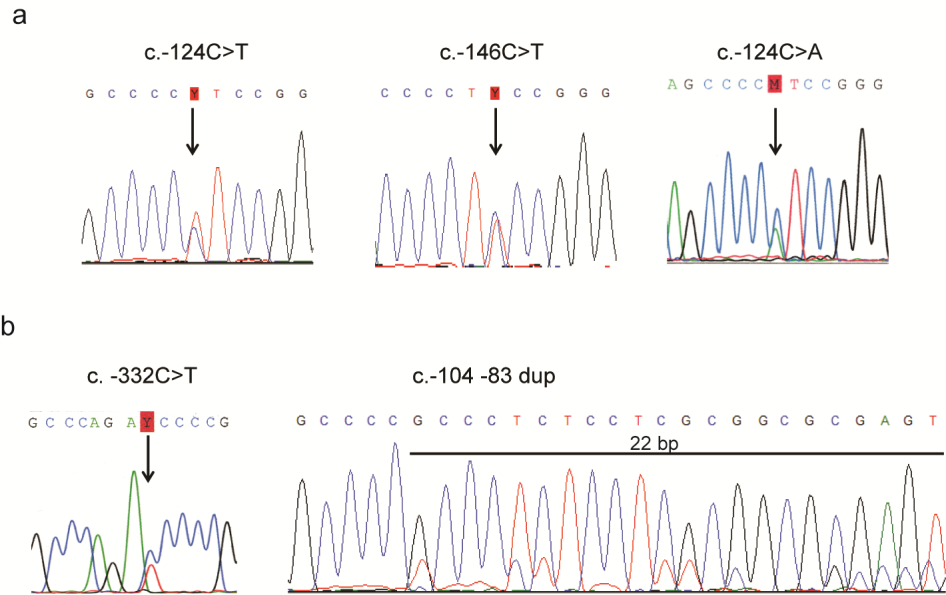


**Figure S1. Direct sequencing of the known and novel mutations of *TERT* promoter in thyroid tumors.** (A) Sequencing chromatographs from representative thyroid tumors harboring the known TERT promoter mutations, c.-124C>T, c.-146C>T, and c.-124C>A by Sanger sequencing analysis. (B) The chromatographs showing sequence for two novel independent *TERT* mutations detected by Sanger sequencing: c.-332C>T in one medullary thyroid carcinoma, and c.-104_-84dup in one papillary thyroid carcinoma. The point mutations are indicated by arrows and the duplication by a bar.

**Table S1. *TERT* promoter polymorphisms in thyroid tumors**

| SNP  **(base change)** | **Position** | **Allele Frequencies*** | **Tumor types positive**  **n/N(%)** |
| --- | --- | --- | --- |
| rs2853669 (T>C) | Chr5: 1295349 | 50% (152/301) | PTC 103/198 (52%) FTC 13/34 (38.2%)  HCC 21/40 (52.5%) PDTC/ATC 9/14 (64.3%)  MTC 6/15 (40%) |
| rs35226131 (G>A)  &  rs35161420 (G>C) | Chr5: 1295373  &  Chr5: 1295452 | 5.6% (17/301) | PTC 11/196 (5.6% )  FTC 2/34 (5.9% )  HCC 1/40 (2.5% )  PDTC/ATC 2/14 (14.3%)  MTC 1/15 (6.7%) |
| rs34233268 (C>G) | Chr5: 1295322 | 0.3% (1/301) | PTC 1/196 (0.5%) |
| rs34764648 (-CG) | Chr5:1295458-1295459 | 0.3% (1/301) | HCC 1/40 (2.5%) |

Abbreviations: SNP, single nucleotide polymorphisms; PTC, papillary thyroid carcinoma; FTC, follicular thyroid carcinoma; HCC, Hürthle cell carcinoma, PDTC/ATC, poorly differentiated/anaplastic thyroid carcinoma; MTC, medullary thyroid carcinoma. *Allele frequencies in this study.

| **Primer** | **Sequence (5’-3’)** |
| --- | --- |
| ***TERT genotyping*** | |
| TERT promoter F1 | GGCCGATTCGACCTCTCT |
| TERT promoter R1 | AGCACCTCGCGGTAGTGG |
| TERT promoter R2 | GGTCCTGGGCGTCTGTG |
| TERT promoter F2 | GTCCTGCCCCTTCACCTT |
| TERT promoter F3 | CACAGACGCCCAGGACC |
| ***Mutagenesis*** | |
| c.-124 C>T_TERT_F | CGTCCCGACCCCTCCCGGGTCCCCGGCCCAGCCCCt  TCCGGGCCCTCCCA |
| c.-124 C>T_TERT_R | GGGCTGGGAGGGCCCGGAAGGGGCTGGGCCGGGG  ACCCGGGAGGGGTCGG |
| c.-124 C>A_TERT_F | CGTCCCGACCCCTCCCGGGTCCCCGGCCCAGCCCCATCCGGGCCCTCCCA |
| c.-124 C>A_TERT_R | GGGCTGGGAGGGCCCGGATGGGGCTGGGCCGGGGACCCGGGAGGGGTCGG |
| c.-146 C>T_TERT_F | CGTCCCGACCCCTTCCGGGTCCCCGGCCCAGCCCCCTCCGGGCCCTCCCA |
| c.-146 C>T_TERT_R | GGGCTGGGAGGGCCCGGAGGGGGCTGGGCCGGGGACCCGGAAGGGGTCGG |
| c.-332 C>T_TERT_F | GAGCGGCGCGCGGGCGGGGAAGCGCGGCCCAGATCCCCGGGTCCGCCCGG |
| c.-332 C>T_TERT_R | GCTCCGGGCGGACCCGGGGATCTGGGCCGCGCTTCCCCGCCCGCGCGCCG |
| c.-104_-83dup_Mut_TERT_F | GCCCTCCCAGCCCCTCCCCTTCCTTTCCGCGGCCCCTCCCCTGCCTTTCAGCGGCCCCG |
| c.-104_-83dup_Mut_TERT_R | CGAGGAGAGGGCGGGGCCGCTGAAAGGCAGGGGAGGGGCCGCGGAAAGGAAGGGGAGGGG |
| rs2853669 _TERT_F | CAGTGGATTCGCGGGCACAGACGCCCAGGACCGCGCTCCCCACGTGGCGG |
| rs2853669 _TERT_R | CCCTCCGCCACGTGGGGAGCGCGGTCCTGGGCGTCTGTGCCCGCGAATCC |
| Primers used for PCR amplification and sequencing of *TERT* promoter. F, forward; R, reverse; ex, exon; EXP, expression. | |

**Table S2. List of primers**

**Table S3. *TERT* promoter mutation analysis in human normal and cancer thyroid cell lines**

| **Cell line** | **Derivation** | ***TERT* promoter mutations** |
| --- | --- | --- |
| TTA1 | Anaplastic thyroid carcinoma | wild-type |
| SW1736 | Anaplastic thyroid carcinoma | c.-124 C>T |
| Htori-3 | Normal human thyroid | wild-type |
| FTC133 | Follicular thyroid carcinoma | c.-124 C>T |
| TPC1 | Papillary thyroid carcinoma | c.-124 C>T |
| K1 | Papillary thyroid carcinoma | c.-124 C>T |
| C643 | Anaplastic thyroid carcinoma | c.-124 C>T |
| T241 | Anaplastic thyroid carcinoma | c.-146 C>T, c.-124 C>T |
